# A critical opinion on adult endogenous neurogenesis as a brain repair mechanism after traumatic brain injury

**DOI:** 10.3389/fnbeh.2025.1543122

**Published:** 2025-02-06

**Authors:** Andrea Aguilar-Arredondo, Angélica Zepeda

**Affiliations:** ^1^IIBBA-CONICET Leloir Institute Foundation, Buenos Aires, Argentina; ^2^Instituto de Investigaciones Biomédicas, Universidad Nacional Autonoma de México, Mexico City, Mexico

**Keywords:** brain plasticity, repair, damage, injury, neurorepair, neurogenesis, functional recovery, sex

Traumatic brain injury (TBI) affects more than 50 million people each year worldwide (Blaya et al., 2022) and leads to diverse functional impairments. Depending on the damaged regions, alterations may compromise different functions, which may recover along time.

Brain responses to injury include events at molecular, cellular and circuit levels (Zepeda et al., 2004; Lim et al., 2014; Kang et al., 2022). However, the study of plastic responses in humans at a subcellular and cellular levels poses many difficulties. Therefore, loss and recovery of functions in humans have been mainly assessed through behavioral evaluation, as well as through neuroimaging and neurophysiological studies. When comparing recovery in humans and in animal models like rodents, we can only suggest the potential contributions of the observed events in some but not all functions (Kozlowski et al., 2013).

Adding up to the complexity of using experimental models of TBI as a proxy to explore nervous recovery in humans, is the difference in the outcomes in male and female clinical and experimental populations. Adult neurogenesis, which has been clearly demonstrated in rats and mice (for a review, see Denoth-Lippuner and Jessberger, 2021), but not beyond doubt in humans (Sorrells et al., 2018; Moreno-Jiménez et al., 2019, for a critical review, see Oppenheim, 2019) has been explored as a potential mechanism subserving functional recovery after brain damage, but it is our opinion that the scientific community is still far from reaching a conclusion regarding its potential role in brain repair.

For the purpose of this opinion, we would like to address four main questions: (1) Are there well established sex-dependent differences in the outcome of TBI in rats and mice that mirror TBI outcomes in humans? (2) To what extent is it possible to extrapolate TBI results obtained in animals to humans? (3) Is neurogenesis a brain recovery mechanism in adult mice and rats? (4) Is neurogenesis a non-debatable mechanism in the adult human brain?

(1) From the total pre-clinical research in animal models in 2016 in Pubmed, only 7% of TBI studies include females and focus on the importance of sex differences for pre-clinical modeling (Späni et al., 2018). Clinical trials as well as experimental observations are mainly based on results from male populations under the argument of how hormonal fluctuations in females may influence the outcome and therefore the results. Thus, the role of hormones in recovery after experimental brain injury has been treated as a “problem” instead of receiving the proper attention to unveil its impact in the process of recovery.

TBI occurs under different circumstances in human males compared to females. In males, TBI is more commonly produced after several types of contact collision or as a result of military combat (Späni et al., 2018); in women TBI mainly results from falls, concussive impacts, and of intimate partner violence (St. Ivany and Schminkey, 2016) and is commonly presented together with multiorgan trauma (Biegon, 2021).

Although sex-related differences in functional outcomes after experimental brain damage is still a matter of debate, there is a body of work that shows that female rats tend to show better outcomes than males, especially after moderate-severe TBI (Gupte et al., 2019). This has been related to increase in hormone levels in the areas surrounding the lesion (for a review see Stein, 2007). However, hormonal variations during the estrous cycle also impact on the results: High levels of progesterone at the time of injury correlate with a better outcome in females, while estrogens may be detrimental for functional recovery (for a review see Stein, 2007).

While hormones have been shown to impact at a molecular and cellular levels including reduction of excitotoxicity, increase in antioxidant responses, synaptogenesis and dendritic arborization (for a review, see Stein, 2001), sex-dependent neurogenesis in the context of damage has been scarcely studied. Actually, to our knowledge there is only one publication (Xiong et al., 2007) which addressed adult neurogenesis after TBI considering the sex of the animals and found that the constitutive number of new neurons did not differ between male and female rats exposed to TBI, while sex-related differences in cell proliferation (but not in neurogenesis) after TBI has been documented (Neale et al., 2023). It is worth mentioning that while sex-related constitutive neurogenesis differences after TBI have not been documented, sexual dimorphisms in response to brain damage in experimental models may become evident after pharmacological treatment and other approaches aimed at promoting recovery (Xiong et al., 2007; Gómez-Porcuna et al., 2024).

The body of evidence regarding sex differences after experimental brain injury ultimately suggest that while several factors may subserve functional recovery in rats, hormonal levels positively impact in the outcome as long as injury occurs after hormonal cycling appears. However, human studies report a worse outcome in females than in males (Gupte et al., 2019; Mikolic et al., 2021) and consider that severity of damage, among other variables, plays a role in the outcome (Mikolic et al., 2021). Thus extrapolating results in experimental TBI to the clinics may not be accurate.

**(2)** Animal models and humans show the capacity to restore altered functions after TBI. However, extrapolation of results from the lab to the clinics needs to consider that evolution after injury depends on several factors such as age of injury, as well as location and type of injury, and it is not always possible to replicate damage in humans with experimental models. Yet many common cell responses that resolve damage involving in particular inflammation-involved mechanisms are shared between experimental and human TBI (for a review, see Liddelow et al., 2024). Also, overcoming several forms of diaschisis (impairments in areas remote from but communicated with the damaged structure) as well as recruitment of regions initially not involved in the performance of altered functions may provide a means for partial recovery (Wiese et al., 2004; Carrera and Tononi, 2014; Boggs et al., 2024). Among responses associated to neuroplasticity rather than to the alleviation of injury in humans as well as in rats is the upregulation of neurotrophic signaling, modulation of neurotransmitters, such as serotonin and GABA and upregulation of hormones (as previously discussed) (McGuire et al., 2019; Shinoda et al., 2021; Fox et al., 2023). In contrast, experimental research results do not yet support the view that new neurons constitutively born in the adult brain serve as a source of functional recovery.

(3) Neurogenesis in the adult brain is a constitutive process in many species (Bonfanti and Amrein, 2018). In mice and rats, resident neural stem cells located in two well defined regions or niches: the subventricular zone of the lateral ventricles and the dentate gyrus of the hippocampus (Lois and Alvarez-Buylla, 1993; Kuhn et al., 1996) proliferate along life and give rise to new neurons through several well described steps (Kempermann et al., 2004; Rasetto et al., 2024).

It has been strongly suggested that new neurons in these species participate in hippocampal-dependent learning and memory, in particular spatial memory and pattern separation (for a review, see Miller and Sahay, 2019). Also, the modulation of the neurogenic process under positive and negative conditions (i.e running vs. inflammation) has been well documented (van Praag et al., 2002; Monje et al., 2003; Perez-Dominguez et al., 2019; for a review see Pérez-Domínguez et al., 2017). However, although neurogenesis after experimental brain damage has been consistently reported to increase (Arvidsson et al., 2002; Aguilar-Arredondo and Zepeda, 2018; Bielefeld et al., 2024) newly-born neurons may not ultimately integrate or survive (Arvidsson et al., 2002; Kang et al., 2022) and their potential role in neurorepair remains unclear.

The pioneering work of Arvidsson et al. (2002), was the first to show that middle cerebral occlusion led to increased neurogenesis in the subventricular zone. New cells acquired a neuronal phenotype and migrated toward the damaged striatum but failed to integrate into the circuit. Since then, several works (Parent et al., 2002; Yamashita et al., 2006; Blaiss et al., 2011; Sun et al., 2015) have evaluated the potential of endogenous and induced neurogenesis in neurorepair after cortical damage, which is mostly affected after TBI and have obtained similar results or have even suggested a detrimental role of the new neurons (Cuartero et al., 2019). Therefore, even when new neurons are generated after cortical damage, there is still a bottle-neck effect that prevents them from significantly participating in repair.

Results after hippocampal damage (which may suffer remote damage after TBI) have shown a different outcome compared to cortical damage and since the work of Nakatomi et al. (2002), other groups including ours have shown that neurons born within the damaged hippocampus in rats have the potential to functionally integrate into the same structure (Aguilar-Arredondo and Zepeda, 2018) but this does not necessarily mean that new neurons replenish the damaged area with new functional neurons nor that new neurons are responsible for functional reorganization. Yet, constitutive neurogenesis could provide a source for reorganization as new neurons may participate in the functional reorganization of a focally damaged circuit.

During adult hippocampal neurogenesis, feedback inhibition of granule neurons increases with physiological maturation (Groisman et al., 2023). After injury, newly generated neurons can become hyperexcitable, potentially leading to post-traumatic epilepsy due to recurrent excitatory synapses from sprouted mossy cells (Butler et al., 2015; Neuberger et al., 2017). Following TBI, feedback inhibition in granule cells increases more than sixfold, driven by excitatory synaptogenesis from new neurons born just before TBI to parvalbumin^+^ (PV) interneurons. Moreover, activation of these new neurons does not contribute to circuit hyperexcitability, suggesting that young granule cells at the time of injury specifically enhance inputs onto PV interneurons (Kang et al., 2022). Overall, while neurons born prior to and around (just before or after) the time of injury may differentially contribute to the excitation/inhibition balance shifts that occur after experimental TBI, their potential role in neurorepair remains inconclusive.

(4) In the human brain, hippocampal adult neurogenesis was first described in 1998 (Eriksson et al., 1998). Since then, many other works have supported this observation. Recent studies, show colocalization of proliferation and neuronal phenotype markers and refer to the presence of new neurons in the adult human hippocampus (for a review see Moreno-Jiménez et al., 2021), but other groups claim a different interpretation of the data (see Alvarez-Buylla et al., 2022). Also, transcriptomic analyses have revealed populations of immature neurons in the dentate gyrus of human brains (Hochgerner et al., 2018; Zhou et al., 2022) but other works have failed to detect them (Cipriani et al., 2018; Sorrells et al., 2018; Franjic et al., 2022). Transcriptomic meta-analysis of different data sets obtained from human brain studies suggest that mouse-based markers may not be the best comparison to analyze neurogenesis in human adult brain given the evolutionary separation between rodents, other primates and humans. Also, doublecortin a cytoskeletal protein of young and migrating neurons, has been used as a proxy to neurogenesis but this protein has been shown to be present in non-neurogenic cells (Klempin et al., 2011; Hagihara et al., 2019). Thus, the presence or absence of only doublecortin, is not enough to argue the existence or the lack of neurogenesis (Tosoni et al., 2023). From the diversity of studies, it has been concluded that new neurons in the human brain have been determined based on rodent neurogenic markers, which is one of the main arguments against the finding of neurogenesis in postmortem human brain studies. The re-analysis of different transcriptomic studies performed by Tosoni et al. (2023), yields the possibility that the controversy of the existence of neurogenesis in the human adult brain could arise from the different methodological, conceptual and biological confounders across studies.

Interestingly, neurons positive for markers associated to a young phenotype have been observed in the human hippocampus (Boldrini et al., 2018; Moreno-Jiménez et al., 2019; Tobin et al., 2019) and even in brains from patients with neurodegenerative diseases. This response has been considered to be an attempt of compensation or early recovery triggered by the disease (Moreno-Jiménez et al., 2019) and supports the evidence showing that a population of neurons in the brain of different species that maintain a “young phenotype” that may activate as a result of a demand, meaning that they are post-mitotic cells that may provide a source for plasticity as they complete maturation given local cues (see Palazzo et al., 2018; Oppenheim, 2019; Jungenitz et al., 2024).

## Discussion

In this opinion article we have resumed some of the findings regarding sex-related differences in in the outcome after TBI in experimental and clinical studies. Female rats tend to have a better outcome than males, while women tend to have worse outcomes. However, after severe TBI, both female rats and women show better outcomes, a topic that deserves close attention. Even when the results obtained in rodents do not necessarily mirror what occurs in humans, it is still important to look at the neurogenic response after brain damage in animal models to widen our view of the multiple processes that accompany repair. Efforts are being conducted to stimulate endogenous neurogenesis as well as to promote reprogramming and integration of cell transplants to promote functional reorganization. It may be a matter of time to understand the potential of neurogenesis as a plastic mechanism involved in human brain repair.
